# An In Vitro Lung System to Assess the Proinflammatory Hazard of Carbon Nanotube Aerosols

**DOI:** 10.3390/ijms21155335

**Published:** 2020-07-27

**Authors:** Hana Barosova, Bedia Begum Karakocak, Dedy Septiadi, Alke Petri-Fink, Vicki Stone, Barbara Rothen-Rutishauser

**Affiliations:** 1 BioNanomaterials Group, Adolphe Merkle Institute, University of Fribourg, 1700 Fribourg, Switzerland; hana.barosova@unifr.ch (H.B.); bedia.karakocak@unifr.ch (B.B.K.); dedy.septiadi@unifr.ch (D.S.); alke.fink@unifr.ch (A.P.-F.); 2 Institute of Experimental Medicine of the Czech Academy of Sciences, 142 20 Prague, Czech Republic; 3 Department of Chemistry, University of Fribourg, 1700 Fribourg, Switzerland; 4 Institute of Biological Chemistry, Biophysics and Bioengineering, Heriot-Watt University, Edinburgh EH14 4AS, UK; v.stone@hw.ac.uk

**Keywords:** lung, in vitro, co-culture, carbon nanotubes, multiwalled carbon nanotubes, air-liquid interface, toxicity, proinflammatory, profibrotic

## Abstract

In vitro three-dimensional (3D) lung cell models have been thoroughly investigated in recent years and provide a reliable tool to assess the hazard associated with nanomaterials (NMs) released into the air. In this study, a 3D lung co-culture model was optimized to assess the hazard potential of multiwalled carbon nanotubes (MWCNTs), which is known to provoke inflammation and fibrosis, critical adverse outcomes linked to acute and prolonged NM exposure. The lung co-cultures were exposed to MWCNTs at the air-liquid interface (ALI) using the VITROCELL^®^ Cloud system while considering realistic occupational exposure doses. The co-culture model was composed of three human cell lines: alveolar epithelial cells (A549), fibroblasts (MRC-5), and macrophages (differentiated THP-1). The model was exposed to two types of MWCNTs (Mitsui-7 and Nanocyl) at different concentrations (2–10 μg/cm^2^) to assess the proinflammatory as well as the profibrotic responses after acute (24 h, one exposure) and prolonged (96 h, repeated exposures) exposure cycles. The results showed that acute or prolonged exposure to different concentrations of the tested MWCNTs did not induce cytotoxicity or apparent profibrotic response; however, suggested the onset of proinflammatory response.

## 1. Introduction

Carbon nanotubes (CNTs) exhibit remarkable features, such as thermal stability, electrical conductivity, and outstanding mechanical durability, and therefore are one of the most widely used nanomaterials (NMs) [1], especially in the fields of energy, electronics, and material composites. The promising potential of CNTs in industrial applications increases the demand for CNT production. However, this high demand is expected to lead to increased contamination of the natural eco-system as well as human exposure, which can potentially occur throughout the NMs life cycle, starting from production to their final disposal. More specifically, the biopersistence and high aspect ratio properties of CNTs are a major concern because of the inhalation of NMs in the workplace during production might induce unwanted pulmonary effects [2,3].

CNTs are made of rolled-up graphene sheets—graphene cylinders, typically limited to a few nanometers in diameter, but their length can range from a few micrometers to millimeters [4]. This unique geometry of relatively small diameter and the enormous length, the needle-like shape, have therefore drawn comparisons with asbestos [5]. There are two main classes of CNTs: single-walled carbon nanotubes (SWCNTs), which consist of one graphene cylinder, and multiwalled carbon nanotubes (MWCNTs) comprises two or more of these graphene cylinders [6].

There has been a vast number of investigations on the potential of MWCNTs released into the environment to induce adverse health effects. In the blood of humans exposed to MWCNT in an occupational setting, aberrant changes in mRNA and ncRNA expression profiles have been reported [7]. A growing number of animal studies demonstrate that exposure to MWCNTs potentially triggers airway injury, inflammation, fibrosis, and granuloma [8,9,10,11]. Among different MWCNTs types, Mitsui-7 MWCNTs (from now on referred to as Mitsui-7) proved to induce a progressive fibrotic response in mice [11,12,13]. Although events leading to fibrosis are part of the normal tissue repair process, pulmonary fibrosis usually refers to a pathological condition where the impaired healing process leads to excessive extracellular matrix production. Pulmonary fibrosis develops as a result of the activation of complex intracellular signaling cascades among multiple cell types, including macrophages, epithelial cells, and fibroblasts [14].

Several in vivo studies have been reported investigating the potentially toxic effects of MWCNTs [15,16,17]. But toxicity evaluation using animals raise scientific, ethical, and financial concerns. In vitro human lung cell models are, on the other hand, widely used to investigate the mechanisms of interactions of exposed particles with the cells [18]. Measurement of the corresponding cellular responses come to prominence as a relatively fast alternative for the primary screening of a broad range of NMs [19,20]. Cell culture models can be used to assess several endpoints related to adverse outcomes of exposure to inhaled substances. Monoculture studies are often performed under submerged conditions using human alveolar epithelial cell line A549 to evaluate the epithelial effects [21,22,23,24]. Direct exposures of fibroblasts to CNTs stimulated progressive cell proliferation or collagen production, the known fibrotic markers [25,26]. Submerged exposure of fibroblasts, epithelial cells, and macrophages used in the presented study was previously used as a first screening tool, and it was shown that exposure to CNTs can induce proinflammatory and profibrotic responses [27,28] An increased proinflammatory response upon repeated exposure to CNT aerosols was also observed in a 3D lung co-culture model (in the presence of immune cells) consisting of cell-lines [29] or primary cells including fibroblasts [30].

When assessing the potential effects of CNTs, equivalent human exposure conditions need to be mimicked by considering exposure methods and occupational dosimetry aspects. Most in vivo studies do not consider the real nature of occupational exposures [31], i.e., the prolonged exposure times and overwhelming high doses. The occupational exposures usually occur in a repeated manner over a longer timeframe in manufacturing facilities, and high doses may, therefore, overwhelm the normal defense mechanisms, resulting in significant initial pulmonary inflammation. On the other hand, the majority of the existing in vitro studies focus on acute exposure using relatively high exposure doses but under submerged conditions [32,33,34]. Submerged conditions are physiologically not relevant since lung cells are exposed to air on their apical surface. To investigate the potential adverse effects of the NMs under relevant and realistic conditions, different air-liquid interface (ALI) exposure equipment were developed previously to aerosolize high-aspect-ratio NMs [35] such as CNTs [36,37,38,39], allowing the homogenous spreading of nebulized material onto the cell surface. Furthermore, such equipment can be supplied with a quartz crystal microbalance (QCM), enabling online measurement of the amount of deposited material.

It is crucial to gain insight into the pulmonary hazard of prolonged (subchronic) CNT exposure at repeated doses that can realistically mimic the in vivo conditions. We recently showed that a lower respiratory tract 3D co-culture model with primary human cells, i.e., EpiAlveolar^™^, could be a promising tool to predict the development of pulmonary fibrosis in response to fibrotic substances such as CNTs [30]. In addition to a proinflammatory and profibrotic response as assessed by cytokine measurements, a disruption in barrier integrity (determined by transepithelial electrical resistance (TEER) measurements) and an increase in alveolar tissue thickening were observed in response to the chemical positive control, transforming growth factor β (TGF-β) [30]. Within the light of this conclusion, the current study was designed to assess the suitability of a 3D lung co-culture model consisting of cell lines (i.e., epithelial cells, macrophages, and fibroblasts) to acute (24 h) and prolonged (96 h) exposures of realistic CNT exposure doses. It is noteworthy that a co-culture model consisting of cell lines can be used for a limited period at ALI due to the issues concerning the overgrowth of the epithelial cells. However, the experiments were designed as a quick screening study to assess the pro-inflammatory and profibrotic cytokine release in response to MWCNT exposure mimicking the occupational exposure conditions.

As a result, this study aimed to expose a 3D lung co-culture model to two different types of MWCNTs, i.e., Mitsui-7 and Nanocyl, as well as silica-based particles as particle controls, i.e., Dörntruper quartz (DQ12) and Min-U-Sil, using a previously developed ALI exposure system at realistic occupational doses while adopting acute (one exposure, sampling after 24 h) and prolonged (five exposures on subsequent days, sampling after 96 h) exposure scenarios. The responsivity of the 3D lung co-culture model as a prescreening tool for possible proinflammatory and profibrotic responses was assessed.

## 2. Results

**Figure 1:**
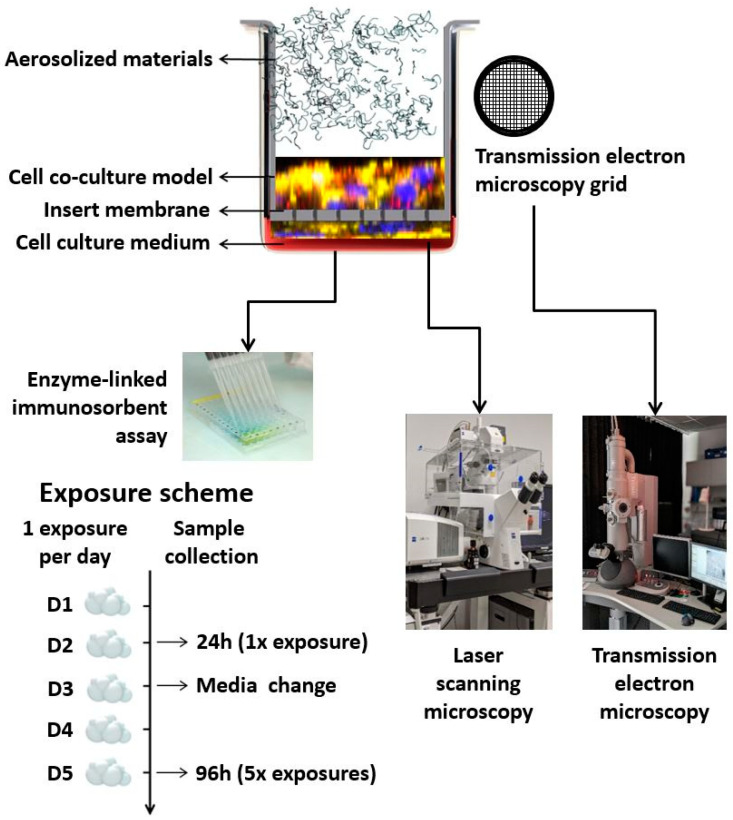
Schematic representation of the experimental setup, exposure scheme, and sample collection.

The scheme of the experimental setup, as well as the exposure scenario and sample collection, are presented in Figure 1.

### 2.1. Material Characteristics

**Figure 2:**
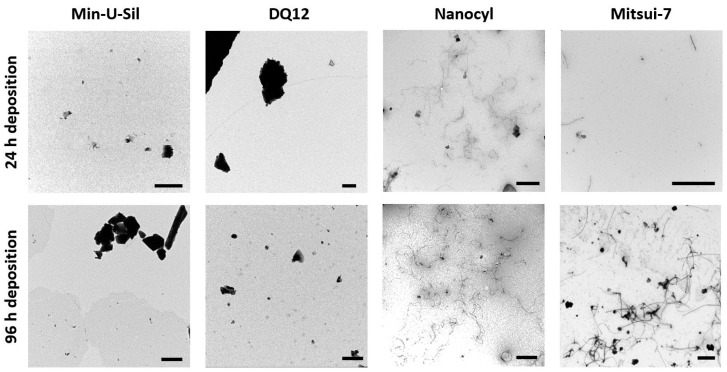
TEM micrographs represent the amounts of 24 h (1 × exposure) and 96 h (5 × exposures) deposition of Min-U-Sil, DQ12, Nanocyl, and Mitsui-7 particles. The scale bar is 1 μm for Min-U-Sil, DQ12, and Nanocyl samples, and 5 μm for Mitsui-7 CNTs.

To estimate the amount and form of test materials deposited on the cells, transmission electron microscopy (TEM) grids were placed in the VITROCELL^®^ Cloud exposure chamber during the material aerosolization and subsequently analyzed using TEM. The daily short-term exposures of the delivered dose, i.e., each exposure took approximately 10 min; as a result, the 24 h time point corresponds to one exposure, while 96 h corresponds to five exposures. In Figure 2, the TEM images present the amounts comparing daily (24 h) deposition, and deposition after 96 h (i.e., five exposures), and the images confirmed that the VITROCELL^®^ Cloud system is a suitable method for executing repeated dose-dependent deposition studies. The representative TEM images of the deposited samples display the morphological differences among the two MWCNTs samples, i.e., Mitsui-7 and Nanocyl. While Mitsui-7 MWCNTs appear as relatively rare singlets or small bundles of thicker tubes, Nanocyl MWCNTs consist of shorter and thinner tangled tubes. The differences in MWCNTs shapes, i.e., stiff straight tubes vs. tangled tubes has resulted in the presence of a multiple number of walls and thus different consequential diameters. On the other hand, Min-U-Sil quartz particles (for further information, see Table 1) with a tendency to form agglomerates and DQ12 particles presenting a homogenous deposition are also shown in Figure 2. Both Min-U-Sil and DQ12 particles are silica quartz particles, with similar particle sizes (< 5 μm), and similar crystallinity. Min-U-Sil possesses approximately 89% crystallinity, while DQ12 present approximately 73% [40].

The differences in material composition are presented in Table 1. The Min-U-Sil consists of pure silica quartz, whereas DQ12 contains approximately 13% impurities. The information about the tested materials, along with the deposited amounts of each material type measured by a QCM device, corresponding to the daily exposure dose, is provided in Table 1. The weekly deposition dose was calculated as five times the daily dose due to the limitation in the number of exposure chambers available. As a result, the cells were exposed to one type of material, adopting one exposure scenario at a time. After each exposure is complete, the instrument is wiped with pre-wetted tissues with ethanol. Subsequently, another exposure experiment is initiated using a different material for the exposure following a different exposure scenario.

### 2.2. Cell Line Co-culture Model

#### 2.2.1. Cytotoxicity and Cell Morphology

**Figure 3:**
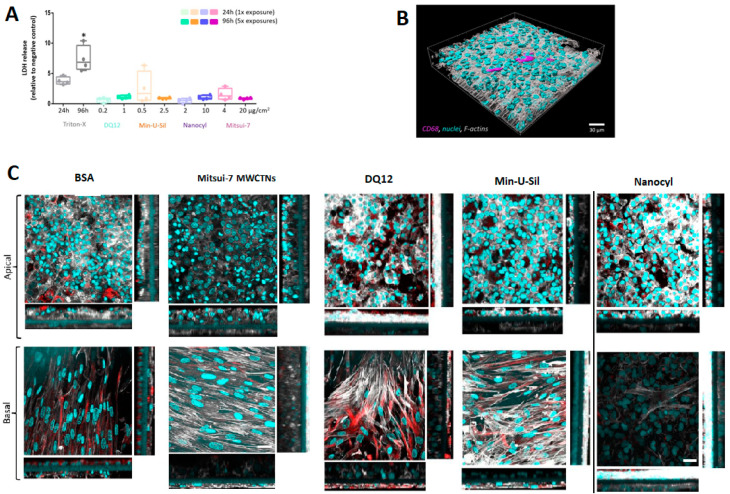
Cytotoxicity and cellular morphology of the 3D co-culture model. (**A**) Cytotoxicity was measured by the LDH assay, data presented in a boxplot with 10–90 percentile. * marks a statistically significant increase compared to the negative control, *n* = 4. (**B**) Representative 3D rendered LSM image of the co-culture model at a 24 h time point, presenting the localization of macrophages within the model. Cyan represents cell nuclei, grey shows cytoskeleton, and magenta stains the macrophages (CD68). (**C**) The LSM images (XY projections) of BSA- and Mitsui-7, DQ12, Min-U-Sil, and Nanocyl-treated samples at 96 h time-point with their corresponding XZ projections showing thickness of the cellular layer, the scale bar is 30 μm. Cyan represents cell nuclei, grey represents cytoskeleton, and red represents vimentin, a type III intermediate filament protein. For 3D rendered LSM images of the co-culture model at 96 h time-point, please refer to the Supplementary Materials, Figures S1–S5. The scale bar is 30 μm.

The amount of lactate dehydrogenase (LDH) released into the cell culture medium was assessed as an important cytotoxicity marker (Figure 3A). The data are presented relative to the negative control (bovine serum albumin, BSA-treated samples). No statistically significant (*p* > 0.05) increase in LDH release was observed for any of the tested materials following the two exposure scenarios. Triton-X (0.2% in phosphate-buffered saline (PBS)) was applied 24 h prior to the sample collection at both time-points as the positive control, which induced a statistically significant (*p* < 0.05) increase of LDH release.

Cell morphology was inspected by laser scanning microscopy (LSM). The cells were labeled for F-actin and cell nuclei. Additionally, the presence of macrophages on the apical side was confirmed by CD68 staining, and the fibroblasts located at the basal side of the insert were stained for vimentin.

The 3D rendered image of the untreated cells at 24 h time-point, showing the location of macrophages on the apical side of the co-culture model, is presented in Figure 3B. Figure 3C shows cell-cultures fixed at 96 h post-exposure to all materials tested. No difference in cell morphology in Mitsui-7 treated cells was observed compared to samples treated with BSA only (negative control). The cells exposed to DQ12 and Min-U-Sil for 96 h exhibited loss of cellular contact and presented irregular shapes, leading to discontinuity and reduced thickness of the epithelial layer. The cell co-cultures treated with Nanocyl MWCNTs are slightly disorganized with discontinuity in the epithelial layer, and a slight increase in the layer thickness of the fibroblasts. Mitsui-7 MWCNTs exposure proved no effect on the fibroblast cell layer thickness. The XZ-projections show overgrowth of the epithelial layer in all samples, regardless of the sample treatment, which proves in low resemblance of in vivo situation at later (96 h) time-point.

#### 2.2.2. Oxidative Stress Response

**Figure 4:**
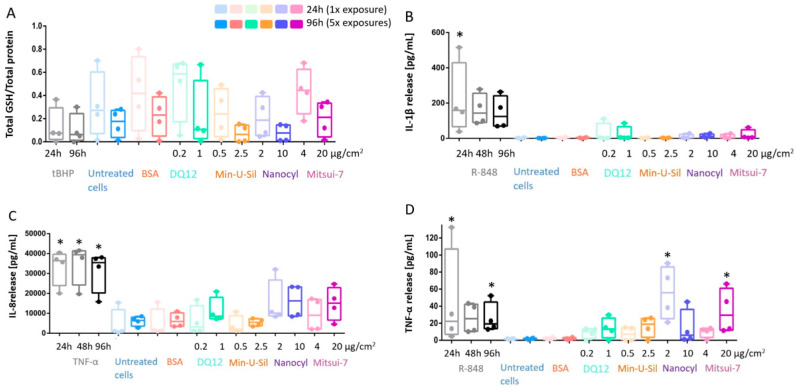
In response to exposure to the tested materials, the extent of oxidative stress and proinflammatory response is assessed via (**A**) GSH content; (**B**) IL-1β; (**C**) IL-8 and (**D**) TNF-α release measurements. Data presented in a boxplot with 10–90 percentile. * marks a statistically significant increase compared to the negative control, *n* = 4.

The decreased glutathione (GSH) content in the cells was reported as an important marker for oxidative stress. The data is presented as the amount of total GSH (intracellular + extracellular) content relative to the total protein measurements. There was no statistically significant decrease in response to any materials tested or at exposure time-points; however, a tendency in the GSH decrease can be seen for Min-U-Sil and Nanocyl exposure results at both 24 h and 96 h time points (Figure 4A).

#### 2.2.3. Proinflammatory Response

The proinflammatory response was assessed by cytokine release measurements via Enzyme-Linked Immunosorbent Assay (ELISA), specifically for Interleukin-1β (IL-1β) and Interleukin-8 (IL-8) detection, as well as for tumor necrosis factor-α (TNF-α) release (Figure 4B–D). No statistically significant increase was detected for IL-1β and IL-8 release in all tested materials at the end of both exposure periods (Figure 4B,C). On the other hand, the increase in TNF-α release was statistically significant (*p* < 0.05) for Nanocyl MWCNTs at 24 h post-exposure, while Mitsui-7 MWCNTs induced statistically significant (*p* < 0.05) release after 96 h post-exposure (Figure 4D). R-848 activates NF-κB, and it was shown that it can trigger IL-1β and TNF-α release in neonates [44]. Therefore, in this study, R-848 was used as a proinflammatory positive control and induced a statistically significant increase after 24 h exposure for IL-1β and TNF-α, and after 96 h for only TNF-α (Figure 4). TNF-α, a potent proinflammatory stimulus [45], was applied as a positive control to induce IL-8 release; a statistically significant (*p* < 0.05) increase of IL-8 release induced by TNF-α compared to negative control was observed for all exposure time points (24 h, 48 h and 96 h) (Figure 4C).

#### 2.2.4. Profibrotic Response

**Figure 5:**
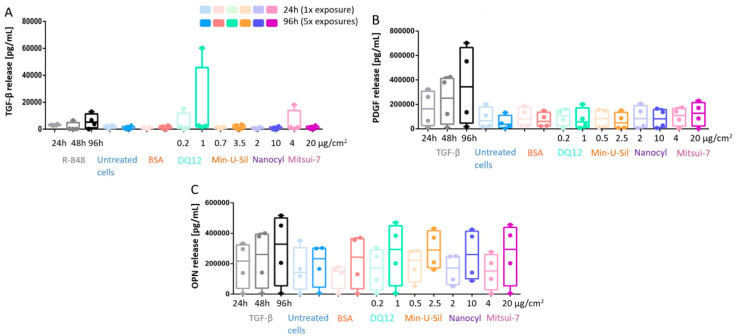
The profibrotic response measurements of the co-culture model. The responses were investigated via (**A**) TGF-β release; (**B**) PDGF release, and (**C**) OPN release. Data presented in a boxplot with 10–90 percentile.

The profibrotic response was assessed by measuring the cytokine release, specifically TGF-β, platelet-derived growth Factor-AA (PDGF), and Osteopontin (OPN), via ELISA (Figure 5). The underlying drive behind the development of an inflammatory response is to clear the foreign material from the tissue and to eventually initiate the repairing pathway, which is facilitated by growth factors, such as the TGF-β. TGF-β was used as a positive fibrotic control because of its involvement in the development of fibrosis in different organs, disturbances of the homeostatic microenvironment, promotion of cell activation, migration, invasion, and excessive extracellular matrix production [30,46]. No statistically significant increase for any of the cytokine release measurements was observed at any exposure time-points upon exposures to any of the tested materials. The overgrowth of epithelial cells may not allow mediators and cytokines to reach fibroblasts and cell culture medium at the amount, which is relevant for activating fibrotic pathway; for instance, lack of TGF-β does not trigger PDGF and OPN release. R-848 was applied as a positive control to induce an increase in TGF-β release (Figure 5A), and TGF-β was also applied as a positive control to induce the PDGF and OPN release (Figure 5B,C), some trends in the increase of cytokine release can be observed for all positive controls at all investigated time points; however, no statistically significant change was detected.

## 3. Discussion

### 3.1. Pros and Cons of the Co-culture Model

This study was designed to mimic the subchronic inhalation of different materials. Previous in vivo studies have shown that the investigated MWCNTs can penetrate deep into the alveolar region of lungs and cumulate in interstitium [11,12]. Macrophages play an essential role in the inflammatory response and surface cleaning (by particle uptake [12]) upon particle exposure and subsequently can play a role in the extent of the fibrotic response. As a result, human alveolar epithelial cells, human lung fibroblasts, and human macrophages were used for designing the present co-culture model. This model follows our previous study [27], where all three cell types cultured as monocultures and were exposed to MWCNTs and silica particles, which are also used in this study. The presented co-culture model is cultured at ALI to provide more realistic culturing conditions. It was previously shown that ALI culturing conditions lead to more enhanced protein release compared to the submerged conditions [47]. In this study, cell lines were used for assembling the presented model, as they represent a relatively stable and cost-effective system while providing experimental flexibility compared to the commercially available primary cells. Although primary cells are unique tools for designing long-term experiments with differentiated cells [30,48], they lack the flexibility needed for co-culturing with other cell types, mainly because they usually require a unique media composition.

Furthermore, primary cells are considered a cost-intensive option for researchers, as they either have to be directly isolated from patients while maintaining high purity of the desired cell population or can be obtained as commercially available models. Although an alveolar model consisting of primary cells is commercially available (EpiAlveolar^™^ [30]), we aimed to present a similar model consisting of cell lines, as a potentially cost-effective tool for enabling rapid pre-screening of particles. This model was designed based on our previous experience with the human alveolar model. We successfully combined human alveolar epithelial cells and human primary immune cells [49] and demonstrated the created model as a powerful tool for screening inflammatory responses upon exposure to various particle types [29,50,51,52]. A summary table (Table 2) is provided to compare the model presented in this study to commercially available 3D lung co-culture models.

### 3.2. Material Concentrations Used for Cell Exposures

The alveolar mass retention during a full lifetime occupational exposure (45 years) period to CNTs of different sizes was modeled and calculated to be in the range of 12.4 to 46.5 μg/cm^2^ [53]. In this study, the maximum deposited concentration after five exposures to Nanocyl was 11 μg/cm^2^ and 20 μg/cm^2^ for Mitsui-7, respectively, which is in the range of the reported lifetime occupational human exposure to MWCNTs [53]. However, it has to be kept in mind that in this study, the amount of CNTs was deposited within five days as the duration of the experiment is the limiting factor for cell line usage. A549 cells are continuously proliferating cells, and therefore they overgrow into a multilayer after 3 to 4 days in ALI, which negatively affects the physiological relevance of the model. On the other hand, the lowest tested exposure concentration (1 μg/cm^2^) for Mitsui-7 and Nanocyl corresponds with concentrations used in mice in vivo [13,54].

Moreover, aerosolization of silica quartz particles resulted in an average deposited concentration up to 1 μg/cm^2^ for DQ12 and 3 μg/cm^2^ for Min-U-Sil after repeated exposures. The tested exposure concentrations correspond to those used in previous in vitro studies, including human lung models, and induced a pro-inflammatory response [29,48]. Furthermore, these concentrations are comparable to in vivo exposure experiments in rats (3–30 mg/rat, which corresponds to 0.6–6 μg/cm^2^, deposition calculated based on [55]) [56].

### 3.3. Oxidative Stress

GSH is known to be essential for several cell processes, mainly interconnected with the maintenance of the thiol-redox status [57]. Therefore, the ratio between the reduced and oxidized forms of GSH is an important indicator of the intracellular redox environment and, at the same time, provides insights regarding cell proliferation, differentiation, and apoptosis [58]. In light of this knowledge, GSH-based oxidative stress measurement assays have been widely implemented [59]. Specifically, GSH measurements in response to exposure to NMs have provided insight regarding the level of the cell’s oxidative stress status [60].

The oxidative stress response upon exposures to silica quartz particles was previously reported in vitro, but not confirmed in vivo [61]. A previous in vitro study showed decreased GSH in fibroblasts 24 h upon exposures to Nanocyl; however, no difference in GSH production was observed 96 h post-exposure compared to the negative control. Min-U-Sil and Mitsui-7 MWCNTs did not induce any significant decrease in GSH either [27]. Similarly, in our study, we did not observe a statistically significant (*p* > 0.05) decrease in GSH levels for any of the tested materials in both exposure scenarios; however, a tendency in the GSH decrease can be observed for Min-U-Sil and Nanocyl exposure results at both time-points (Figure 4A). This finding is in line with the proteomics investigation of the presented co-culture response upon exposures to Mitsui-7, where no response was observed upon exposure to Mitsui-7 compared to the negative control [47].

### 3.4. Proinflammatory Response

A proinflammatory cytokine is a type of signaling molecule secreted from certain cell types and promotes inflammation in response to foreign materials, in this case, MWCNTs. Among them, IL-1β, IL-8, and TNF-α are known to play a vital role in mediating the innate immune response and therefore considered biomarkers of nanoimmunotoxicity [62].

Certain particle types have been shown to induce immunotoxicity. Among them, DQ12 is well studied, and have been recruited in in vitro studies as a potent proinflammatory stimulus [29,63]. On the other hand, Min-U-Sil was found to induce increased IL-1β release in macrophage monocultures (differentiated THP-1), but no response in fibroblasts or epithelial cells was reported [27]. In vivo experiments with mice showed a strong inflammatory response upon acute exposure to a high dose of Min-U-Sil [64]. Nanocyl and Mitsui-7 MWCNTs did not induce any proinflammatory response in epithelial cells, fibroblasts, or macrophage monocultures in vitro [27]. Although individual in vitro studies assessing the potential toxicity of MWCNTs of interest did not report proinflammatory responses, it should be noted that these studies were either using monocultures of cells or the exposure conditions were not representative, i.e., submerged conditions as opposed to creating an ALI prior to the exposure.

In this study, we have adapted two exposure scenarios, as explained in the Methods section. The results indicated statistically significant changes in TNF-α secretion levels in response to two different exposure scenarios (acute (24 h) vs. prolonged (96 h)) for both MWCNT types tested. A significant elevation in TNF-α levels for Nanocyl at 24 h post-exposure was detected. In comparison, Mitsui-7 MWCNTs induced a statistically significant (*p* < 0.05) increase in TNF-α levels 96 h post-exposure (Figure 4D). TNF-α levels specifically play a vital role in proinflammatory response assessment because it is expressed in the early stages of cell inflammation [65]. Therefore, it can be concluded that in a realistic occupational exposure scenario, exposure to MWCNTs does initiate cell inflammation and eventually can result in cell death.

### 3.5. Profibrotic Response

Exposure to NMs is known to play a role in the development of chronic pulmonary diseases, especially fibrosis [66]. Among all NMs, MWCNTs specifically pose a danger because relatively large quantities are being used in numerous manufacturing practices, and thus will inadvertently lead to human exposure. Therefore, there have been continuous efforts to evaluate the potential link between fibrosis and MWCNTs exposure. It was previously shown that both Mitsui-7 [12,67] and Nanocyl [42] exposures lead to the development of pulmonary fibrosis in rodents. However, it was reported that Nanocyl caused less upregulation of genes involved in fibrosis development compared to long and thick MWCNTs [42]. These results are consistent with an in vitro study investigating epithelial cell (A549), macrophage (differentiated THP-1), and fibroblast (MRC-5) monocultures. Mitsui-7 exposures led to profibrotic response (TGF-β release), and OPN releases in epithelial cells. On the other hand, Nanocyl increased only TGF-β release from fibroblasts [27], which has previously been used in vitro to stimulate the pro-inflammatory response [35,48,63] and in vivo to stimulate the development of pulmonary fibrosis [56,68].

In this study, we did not observe a statistically significant increase in TGF-β, PDGF, or OPN release (Figure 5). Interferon-γ (1 μg/mL) was also used as a positive control to stimulate TGF-β, PDGF, and OPN release (data not shown), but no increase in profibrotic mediators release was detected. In addition to the evaluation of profibrotic mediator releases, we also measured fibrotic markers, the collagen type I and fibronectin release, but did not detect a statistically significant increase in both indicator release levels (data not shown), although this combination of investigated endpoints proved its suitability in predicting profibrotic response in vitro earlier [30]. Furthermore, the use of the Sircol^TM^ soluble collagen assay to detect acid and pepsin soluble collagen was considered; however, the presence of serum in the cell culture medium posed interference on the assay results; therefore, the use of this assay was eliminated from the study. It is also noteworthy to state that at the cellular level, proinflammatory mediator detection is considered one of the key events (KE) in the adverse outcome pathway (AOP) for fibrosis [30]. The presented model resembles the alveolar region of the human lungs, and the concept is similar to the EpiAlveolar^TM^ model used in our previous study [30]. Both models use human lung epithelial cells, fibroblasts, and macrophages, while the EpiAlveolar^TM^ model additionally includes endothelial cells. However, the main differences of the two models are the cells used: cell lines vs. primary cells, and the duration of the experiment, i.e., the length of the periods while each cell model remains stable throughout the exposures. For the EpiAlveolar^TM^ model, it was possible to perform exposures over three weeks at ALI, whereas the cell line model only is stable for 3–4 days as the A549 cells start to overgrow at ALI. When comparing the data from both studies, the model with primary cells is more suitable for such an investigation to assess repeated and long-term effects, as it is capable of predicting profibrotic response upon exposure to TGF-β and other NMs. On the other hand, cell line models still offer a cheaper and easy-to-handle option for hazard prescreening of NMs to assess cytotoxicity and inflammatory markers. Finally, the proposed model is not limited to testing potentially hazardous nanomaterials. It can also be implemented to test anti-inflammatory [69,70] and anti-fibrotic [71] nanoformulations in the field of nanomedicine.

## 4. Materials and Methods

### 4.1. Chemicals and Reagents

All chemicals and reagents used were obtained from Sigma-Aldrich (Buchs, Switzerland) unless stated otherwise.

### 4.2. Sample Preparation

BSA solution (0.1% in ultrapure H_2_O) was sterile filtered (0.2 μm pore size; Nalgene, Thermo Scientific, MA, USA). BSA was used as a dispersant for MWCNTs. Mitsui-7 MWCNTs (MWCNTs-7; Mitsui & Co, Tokyo, Japan) and Nanocyl-7000 MWCNTs (referred to as Nanocyl; Nanocyl S.A., Sambreville, Belgium; received from European Commission Joint Research Centre, Ispra, Italy) were dispersed in BSA solution. Briefly, pre-weighted dry MWCNTs powder was heat sterilized at 100 °C overnight, left to cool down, and subsequently 0.1% BSA solution was added to obtain a stock solution with the concentration of 50 μg/mL. This suspension was sonicated for 3 h with continuous shaking to disperse the MWCNTs and subsequently stored at 4 °C until further use.

Two different types of silica quartz particles were used as reference materials. Min-U-Sil is an inert, high purity white crystalline silica recently reported as a potential profibrotic agent [28,72], while Dörntruper quartz (DQ12; composed of 87% crystalline silica and amorphous silica with kaolinite impurities) was reported as a proinflammatory agent in vitro [73], as well as profibrotic agent in vivo [56,74,75]. Both silica materials were dispersed in ultrapure sterile filtered H_2_O at a concentration of 100 μg/mL.

All stock suspensions were sonicated for 1 h prior to the exposure experiments.

### 4.3. Material Characterization

#### 4.3.1. Electron Microscopy

To investigate the deposition of aerosolized materials, transmission electron microscopy (TEM) 300 mesh carbon-coated copper grids were placed into the exposure chamber prior to the experiment.

TEM grids with deposited material were used without any further treatment. Representative images were captured using a TEM (FEI Tecnai Spirit, Hillsboro, OR, USA) operating at 120 kV and equipped with a Veleta CCD camera (Olympus, Japan).

#### 4.3.2. Endotoxin Content

The endotoxin concentration in the MWCNTs suspensions was measured using the Pierce^TM^ LAL Chromogenic Endotoxin Quantitation Kit (ThermoFisher Scientific, Basel, Switzerland), following the manufacturer’s instructions and all suspensions were below 0.5 EU/mL.

### 4.4. Human Cell Line Co-Culture Model

All cell lines—human fibroblasts MRC-5, human alveolar epithelial cells type II A549 and human monocytes THP-1 were purchased from American Type Culture Collection (ATCC, Manassas, VA, USA) and cultivated according to the ATCC protocol prior assembled into co-culture model.

Human epithelial cells type II (A549 cell line) were cultured in Roswell Park Memorial Institute medium (RPMI 1640) supplemented with 10% Fetal Bovine Serum (FBS), 1% Penicillin/streptomycin and 1% L-Glutamine (all stated chemicals were obtained from Gibco, Gaithersburg, MD, USA). Human monocytes (THP-1 cell line) were cultivated in supplemented RPMI 1640, as mentioned above, with extra 0.05 mM β-mercaptoethanol. To differentiate monocytes into macrophages, prior to co-culture assembling, the THP-1 cells had to be activated by Phorbol 12-Myristate 13-Acetate (PMA; 20 ng/mL in supplemented medium without β-mercaptoethanol) overnight at the density of 4 × 10^5^ cells/mL. Human fibroblasts were cultivated in Minimum Essential Medium (MEM) supplemented with 10% FBS, 1% Penicillin/streptomycin, 1% L-Glutamine, and 1 × Non-Essential Amino Acids (all Gibco, Gaithersburg, MD, USA).

The co-culture model was composed by following the next steps. The Transwell^®^ 12-well inserts (surface area of 0.9 cm^2^, pores of 0.4 μm diameter, PET membranes; BD Biosciences, Allschwil, Switzerland) were inverted and placed into a sterile petri dish. Then the MRC-5 cells were seeded on the basolateral part of the insert at the density 10^4^ cells/cm^2^ and incubated for 3 h at 37 °C, 5% CO_2_ to fully attach to the insert membrane. After the incubation period, inserts were turned back and placed into the 12-well plate containing 1.5 mL supplemented MEM. Subsequently, A549 cells were seeded on the apical side of the insert at the density of 2.8 × 10^5^ cells/cm^2^ with 1 mL of supplemented RPMI 1640 and incubated for 4 days (d) at 37 °C, 5% CO_2_. After the 4-d incubation period of A549-MRC-5 co-cultures, differentiated THP-1 in supplemented RPMI 1640 were seeded on the top of A549 cells at the density of 28 × 10^3^ cells/cm^2^. The plates were then incubated for 2 d at 37 °C, 5% CO_2_, and subsequently lifted to ALI 24 h prior to the experiment by removing the medium from the apical side and replacing medium from the basolateral side with 0.6 mL of the medium mixture (RPMI:MEM, 1:1).

### 4.5. The Air-liquid Interface (ALI) Cell Exposure Method

Co-cultures were exposed at the ALI using the VITROCELL^®^ Cloud system (Waldkirch, Germany). Briefly, the exposure system consists of a nebulizer, an exposure chamber as well as a QCM (operated at 5 MHz, detection limit: 0.1 μg/cm^2^), allowing to measure and record the deposited dose online. For each aerosolization experiment, 200 μL of sample’s stock solution with 2 μL of 0.09% NaCl (NAAPREP^®^ physiological saline, GlaxoSmithKline, Evreux, France) was added to the nebulizer (Aeroneb^®^ Lab, Dangal, Galway, Ireland; mesh size 10 μm for MWCNTs, 4–6 μm for Min-U-SIl and 2.5–6 μm for DQ12). The vibrating perforated membrane at the neck of the nebulizer generates the aerosols and diverts them into the exposure chamber. Inside the chamber, the aerosols in the cloud gently deposit onto the cell surfaces that are maintained at the ALI. The flow rate of nebulizer (the flowrate is preset by the instrument provider, cannot be controlled) was ideal for the aerosols to sufficiently mix within the entire chamber, hence resulting in uniform droplet deposition. VITROCELL^®^ Cloud exposure chamber was wiped with pre-wetted ethanol tissues prior to each exposure experiment.

### 4.6. Exposure Scenarios

The biological response of the co-culture model was assessed following exposure to all tested materials at the doses presented in Table 1. Two different exposure scenarios were performed, i.e., acute (24 h) exposure and prolonged (96 h) continuous exposure. The cells were exposed apically at the ALI daily and maintained at 37 °C in 5% CO_2_ throughout each exposure scenario. The cells were exposed to one dose of the material at the acute exposure scenario. In contrast, the cells following the prolonged exposure scenario were exposed to five times the material dose on subsequent days for 96 h in total.

### 4.7. Biochemical Analysis

#### Cytotoxicity

The LDH release into the supernatant as a result of cell membrane rupture is a well-known indicator of cytotoxicity. The amount of LDH release was evaluated using a commercially available LDH diagnostic kit (Roche Applied Science, Mannheim, Germany), according to the manufacturer’s protocol. Each sample was tested in triplicates; the enzyme activity was measured photometrically. The absorbance was measured at 490 nm with a reference wavelength of 630 nm. LDH values are presented relative to the negative control (BSA-treated cells). Cell cultures exposed apically to 0.2% Triton X-100 for 24 h were used as a positive control.

### 4.8. Cell Morphology

The cell morphology was evaluated using laser scanning microscopy. At the respective time-points, the cell cultures were fixed for 15 min in 4% paraformaldehyde in PBS at room temperature, subsequently washed 3 × with PBS and stored in PBS at 4 °C until further immunofluorescence staining could occur.

Prior to the immunofluorescence staining, samples were treated with 0.1 M glycine for 15 min and subsequently permeabilized with 0.2% Triton X-100 for another 15 min, both at room temperature. All antibodies were diluted in 0.3% Triton X-100 and 1% BSA in PBS and incubated for 2 h. The cells were first stained with primary antibodies (dilution 1:100) and subsequently with secondary antibodies, 4’,6-diamidin-2-fenylindol (DAPI) and rhodamine-phalloidin (dilution 1:100, Molecular Probes, Eugene, OR, USA). Specifically, anti-CD68 and chicken anti-vimentin were used as the primary antibodies and anti-mouse Alexa 488, anti-chicken Alexa 647 (Abcam, Cambridge, MA, USA), phalloidin-rhodamine and DAPI were used as the fluorophores. Anti-CD68 is used for macrophage staining, anti-vimentin stains intermediate filaments of the fibroblasts, phalloidin-rhodamine stains F-actin cytoskeleton and DAPI stains nucleus. Following antibody incubation, cell culture inserts were embedded in Glycergel (DAKO, Santa Clara, CA, USA). Cell morphology was evaluated by visualization of samples via an inverted laser scanning confocal microscope (LSM) 710 (Axio Observer.Z1, Carl Zeiss, Oberkochen, Germany). Image processing was conducted with IMARIS 3D restoration software (Bitplane AG, Zurich, Switzerland).

### 4.9. Oxidative Stress

The total amount of reduced intracellular GSH in the cell cultures was quantified using a GSH assay kit (following supplier’s protocol, Cayman Chemical Company, Ann Arbor, MI, USA) as a well-known marker for oxidative stress. Levels of oxidative stress are presented as a ratio of total GSH to the total amount of protein of each sample. Total protein content was measured by the Pierce bicinchoninic acid (BCA) protein assay kit (Pierce Protein Research Products, Thermo Scientific, Rockford, IL, USA) according to the manufacturer guidelines. Tert-Butyl Hydrogen Peroxide (tBHP) at a concentration of 135 mM acted as the positive assay control.

### 4.10. Cytokine Secretion

#### 4.10.1. Proinflammatory Response

The IL-1β, TNF-α, and IL-8 secretion were assessed using the commercially available DuoSet^®^ ELISA Development diagnostic kit (R&D Systems, Zug, Switzerland), according to the manufacturer’s protocol. Cells treated apically with 1 μg/mL of TNF-α (ImmunoTools, Friesoythe, Germany) served as the positive control for IL-1β (data not shown) and IL-8 assays. R-848 applied apically onto the cell surface at 6 μg/mL was used as a positive control for IL-1β, TNF-α, and IL-8 (data not shown) assays. All positive controls were tested at 24 h, 48 h, and 96 h post-exposure time-points in both co-culture systems.

#### 4.10.2. Profibrotic Response

The TGF-β, PDGF, and osteopontin (OPN) release into the supernatant of exposed cells was quantified using the respective ELISA DuoSet^®^ Development diagnostic kit (R&D Systems, Zug, Switzerland), following the manufacturer’s protocol. Based on the supplier’s protocol, pH activated form of TGF-β was assessed (the activation occurs straight prior to the assay). Cell cultures exposed apically to 1 μg/mL of Interferon-gamma (IFN-γ) were used as a positive control for TGF-β assay (data not shown), while apical cell treatment with 100 ng/mL TGF-β served as the positive control to induce PDGF and OPN release. R-848 applied apically onto the cell surface at 6 μg/mL was used as a positive control for the TGF-β assay. All positive controls were tested at 24 h, 48 h, and 96 h post-exposure time-points in both co-culture systems.

### 4.11. Statistical Analysis

For each data point, four independent experiments were performed (four biological replicates), one technical replicate per treatment was prepared; each endpoint was measured in triplicates. Statistical analysis was performed using GraphPad Prism 6 (GraphPad Software Inc., La Jolla, CA, USA) software. A parametric one-way analysis of variance (ANOVA) with subsequent Dunnett test was performed. Results were considered significant if *p* < 0.05.

## 5. Conclusions

In conclusion, our in vitro co-culture model consisting of three human cell lines relevant to assess inflammation and fibrosis showed a proinflammatory response upon exposure to the positive controls, and a statistically significant TNF-α release was detected after exposure to both MWCNTs tested. On the other hand, no cytotoxicity or profibrotic response was observed in response to exposure to both types of MWCNTs. While the presented model is not suitable to predict the profibrotic response at the 96 h time point, it can be used for both acute (24 h) and prolonged (96 h) proinflammatory response investigations. As a result, our representative 3D lung co-culture model can be used to assess the proinflammatory response to MWCNTs aerosols; if the results induce a positive response, then further investigations involving primary cells allowing a repeated exposure up to several weeks are recommended. Finally, it is important to note that our model is not limited to testing potentially hazardous nanomaterials. A future study is underway to harness this representative lung system to test anti-inflammatory and anti-fibrotic nanoformulations.

## Figures and Tables

**Table 1:** Information about tested materials, daily deposited dose was measured with a quartz crystal microbalance (QCM), the 96 h deposited doses were calculated as five times the 24 h dose.

Material	Type of the Material	Particle Size (μm) (Length)	Particle Size (μm) (Diameter)	Dispersant	24 h Dose (μg/cm^2^)	96 h Dose (μg/cm^2^)
DQ12 [41]	Quartz sand (87% crystalline silica + amorphous silica, kaolinite)		≤ 5	Ultrapure water	0.18 ± 0.04	0.90 ± 0.22
Min-U-Sil [41]	Crystalline silica		≤ 5	Ultrapure water	0.53 ± 0.17	2.66 ± 0.86
Nanocyl [42]	MWCNTs	~ 1.5	~ 0.01	0.1% BSA	2.33 ± 0.87	11.67 ± 4.34
Mitsui-7 [43]	MWCNTs	~ 13	~ 0.05	0.1% BSA	3.93 ± 0.95	19.65 ± 4.76

**Table 2:** The comparison of the 3D lung co-culture model presented in this study to commercially available 3D lung co-culture models consisting of primary cells.

Property	The Presented Model in This Study	Commercially Available Models Consisting of Primary Cells [30,48]
Ease of assembling the model	Easy	Sold as ready-to-use
Adjusting/ tuning based on desired investigation endpoints	Possible	Not possible
Inclusion of immune cells	Yes	No *
Possibility to use classical cell culture media	Yes	No
Overall cost	Low	High
Suitability to be used for long-term experiments	No	Yes
Barrier tightness (trans-epithelial electrical resistance)	Low	High

* Can be included by the end-user, but requires an extensive optimization step as immune cells are cultured in a different and complex cell media as opposed to commercially available models.

## Supplementary Materials

Supplementary materials can be found at https://www.mdpi.com/1422-0067/21/15/5335/s1. Figure S1. 3D rendered LSM image of the co-culture model at 96 h time-point exposed to BSA. Figure S2. 3D rendered LSM image of the co-culture model at 96 h time-point exposed to Mitsui-7 MWCNTs. Figure S3. 3D rendered LSM image of the co-culture model at 96 h time-point exposed to DQ12. Figure S4. 3D rendered LSM image of the co-culture model at 96 h time-point exposed to Min-U-Sil. Figure S5. 3D rendered LSM image of the co-culture model at 96 h time-point exposed to Nanocyl.

## Abbreviations

3D	Three dimensional
ANOVA	Analysis of variance
ATTC	American type culture collection
BCA	Bicinchoninic acid
BSA	Bovine albumin serum
CCD	Charged coupled device
CD	Cluster of differentiation
CNT	Carbon nanotubes
DAPI	4′,6-diamidino-2-phenylindole
ELISA	Enzyme-linked immunosorbent assay
FBS	Fetal bovine serum
GSH	Glutathione
IL	Interleukin
LDH	Lactate dehydrogenase
LSM	Laser scanning microscopy
MEM	Minimum essential medium
MWCNT	Multiwalled carbon nanotubes
NM	Nanomaterial
OPN	Osteopontin
PBS	Phosphate buffered saline
PDGF	Platelet-derived growth factor
PET	Polyethylene terephthalate
PFA	Paraformaldehyde
PMA	Phorbol 12-myristate 13-acetate
QCM	Quartz crystal microbalance
RPMI	Roswell Park Memorial Institute
SEM	Standard error of the mean
tBHP	Tert-butyl hydrogen peroxide
TEER	Transepithelial electric resistance
TEM	Transmission electron microscopy
TGF	Transforming growth factor
TNF	Tumor necrosis factor
