# Supplementary material for: An In Vitro Lung System to Assess the Proinflammatory Hazard of Carbon Nanotube Aerosols
Source: Int J Mol Sci. 2020 Jul 27;21(15):5335. doi: 10.3390/ijms21155335 (PMC7432093; doi:10.3390/ijms21155335)
Supplement: Supplementary file 1 [file ijms-21-05335-s001.pdf]

Supplementary Information for

An *In Vitro* Lung System to Assess the Proinflammatory Hazard of Carbon Nanotube Aerosols

Hana Barosova [1,2], Bedia Begum Karakocak [1], Dedy Septiadi [1], Alke Petri-Fink [1,3], Vicki Stone [4] and Barbara Rothen-Rutishauser [1,*]

[1] BioNanomaterials Group, Adolphe Merkle Institute, University of Fribourg, Fribourg 1700, Switzerland; hana.barosova@unifr.ch (H.B.); bedia.karakocak@unifr.ch (B.B.K.); dedy.septiadi@unifr.ch (D.S.); alke.fink@unifr.ch (A.P.F.)

[2] Institute of Experimental Medicine of the Czech Academy of Sciences, Prague 142 20, Czech Republic

[3] Department of Chemistry, University of Fribourg, Fribourg 1700, Switzerland

[4] Institute of Biological Chemistry, Biophysics and Bioengineering, Heriot-Watt University, Edinburgh, EH14 4AS, UK; v.stone@hw.ac.uk (V.S.)

* Correspondance: barbara.rothen@unifr.ch; Tel.: +41-26-300-9502

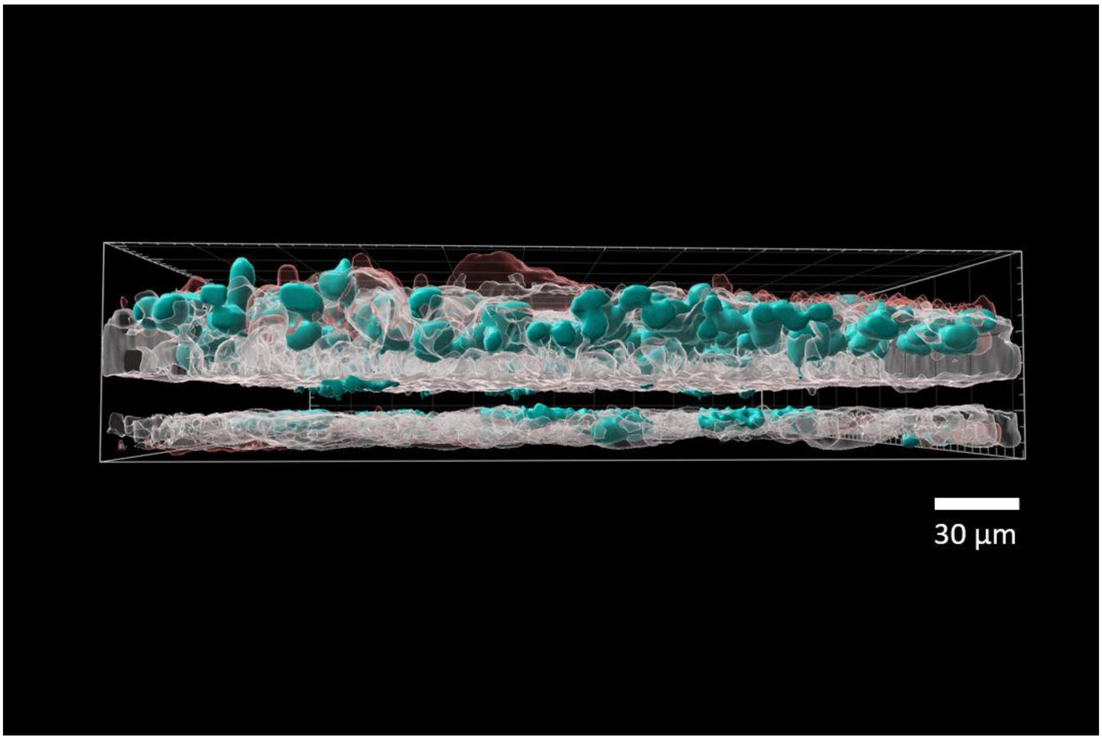

Figure S1. 3D rendered LSM image of the co-culture model at 96 h time-point exposed to BSA. Cyan represents cell nuclei, grey represents cytoskeleton, and red represents vimentin, a type III intermediate filament protein.

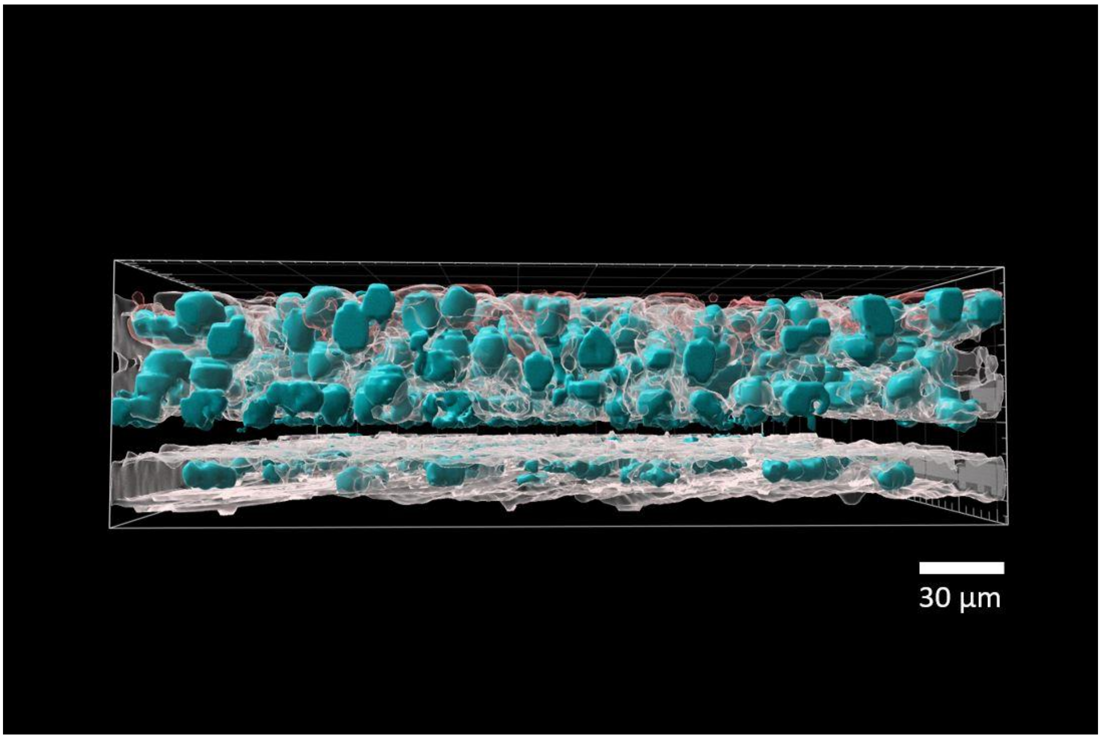

Figure S2. 3D rendered LSM image of the co-culture model at 96 h time-point exposed to Mitsui-5 MWCNTs. Cyan represents cell nuclei, grey represents cytoskeleton, and red represents vimentin, a type III intermediate filament protein.

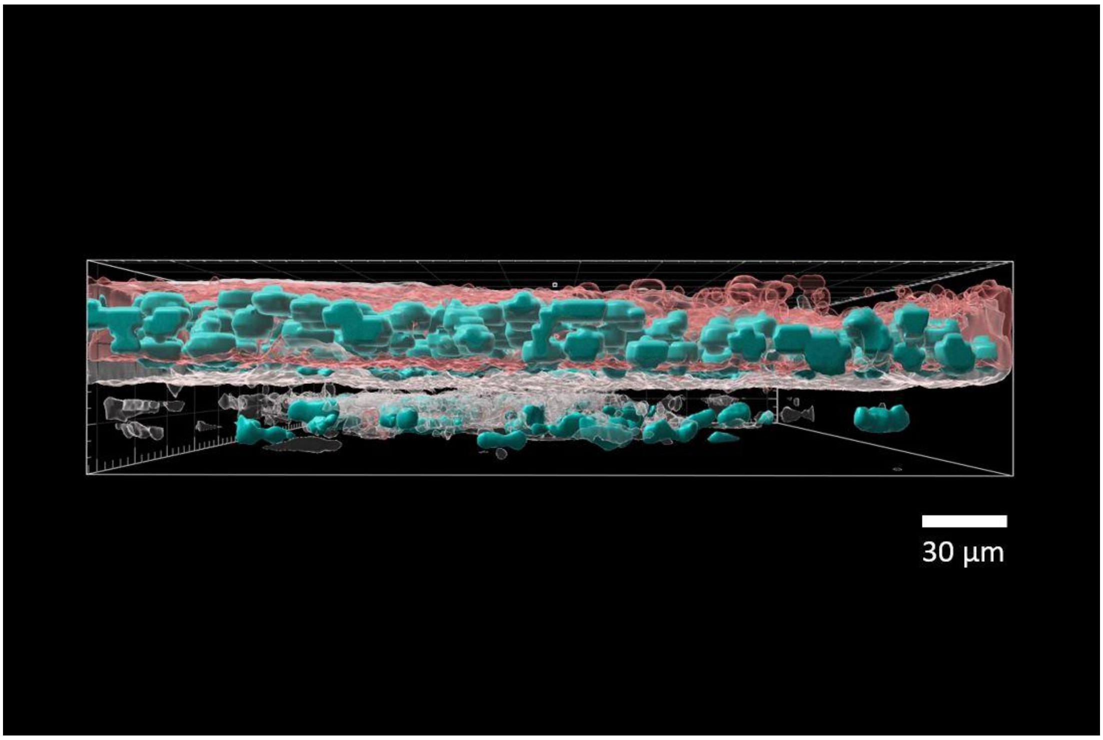

Figure S3. 3D rendered LSM image of the co-culture model at 96 h time-point exposed to DQ12. Cyan represents cell nuclei, grey represents cytoskeleton, and red represents vimentin, a type III intermediate filament protein.

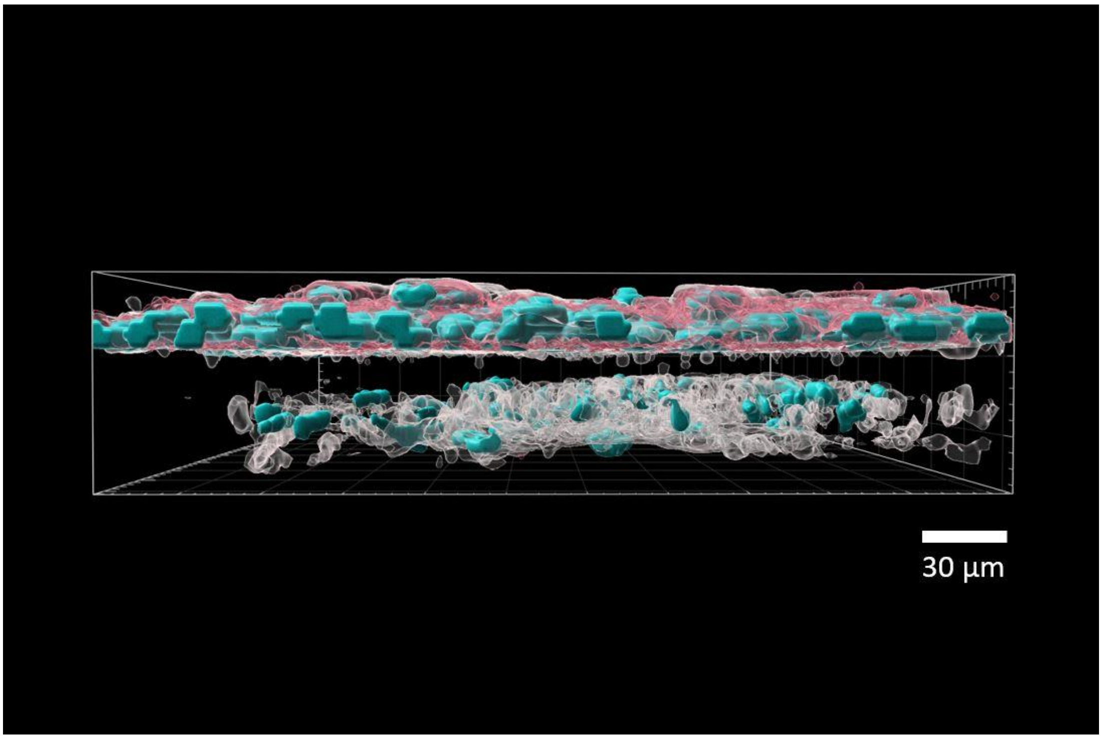

Figure S4. 3D rendered LSM image of the co-culture model at 96 h time-point exposed to Min-U-Sil. Cyan represents cell nuclei, grey represents cytoskeleton, and red represents vimentin, a type III intermediate filament protein.

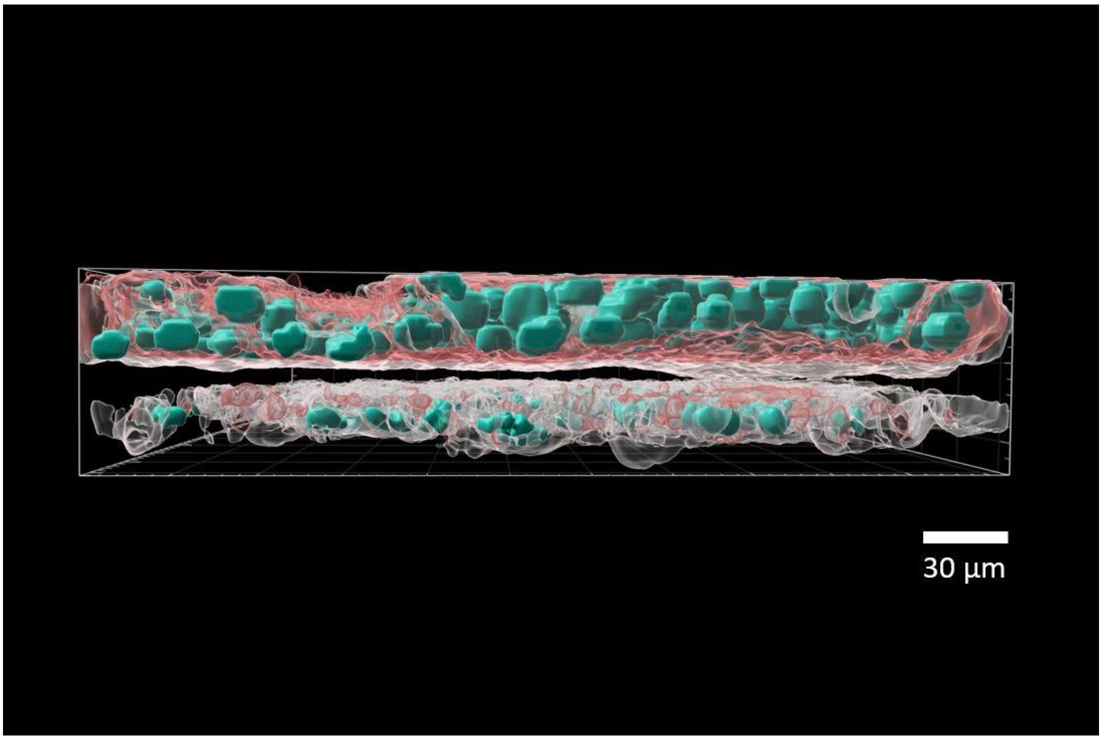

Figure S5. 3D rendered LSM image of the co-culture model at 96 h time-point exposed to Nanocyl. Cyan represents cell nuclei, grey represents cytoskeleton, and red represents vimentin, a type III intermediate filament protein.
